# Delayed diagnosis of pituitary LCH with BRAFV600E mutation: A case report and literature review

**DOI:** 10.1097/MD.0000000000045274

**Published:** 2025-10-17

**Authors:** Yiwei Hou, Yu Yang, Xiangyu Liao, Li Yi, Shixin Li, Beihan Li, Yunxi Fu, Zijun Zhou, Mingxu Tong, Wufei Zhu

**Affiliations:** a The First College of Clinical Medical Science, China Three Gorges University, Yichang, Hubei Province, China; b Department of Endocrinology, Yichang Central People’s Hospital, Yichang, Hubei Province, China; c Department of Hepatobiliary Surgery, Yichang Central People’s Hospital, Yichang, Hubei Province, China; d Department of Oncology, Yichang Central People’s Hospital, Yichang, Hubei Province, China; e Medical Technology College of Qiqihar Medical College, Qiqihar, Heilongjiang Province, China; f Basic Medical College of Qiqihar Medical University, Qiqihar, Heilongjiang Province, China.

**Keywords:** BRAF V600E mutation, case report, central diabetes insipidus, chemotherapy, langerhans cell histiocytosis

## Abstract

**Rationale:**

This case report highlights the diagnostic challenges and clinical complexities of adult-onset pituitary Langerhans Cell Histiocytosis (LCH), a rare entity with a prolonged and indolent progression. This underscores the critical need for heightened clinical suspicion in patients with chronic endocrine dysfunction, particularly in those with multisystem comorbidities. The atypical 10-year diagnostic odyssey coupled with molecular confirmation of the BRAFV600E mutation adds novel insights into delayed recognition and genetic markers in adult LCH, addressing gaps in the existing literature on protracted pituitary involvement.

**Patient concerns:**

A 38-year-old woman experienced decade-long polydipsia, polyuria, fatigue, irregular menstruation and progressive panhypopituitarism. Laboratory findings showed hypernatremia (Na 155.7–157.5 mmol/L), hyperchloremia (Cl 113.6–119.1 mmol/L), low TSH (0.015–0.144 µIU/mL) with low-to-normal FT3/FT4, persistently low cortisol, low FSH/LH, and low PRL. MRI revealed a 1.9 × 1.4 × 1.2 cm pituitary mass.

**Diagnosis:**

Pituitary LCH was confirmed by nasal saddle area biopsy (S100/CD1a positivity) and BRAFV600E mutation detection via ARMS-PCR.

**Interventions:**

Management included desmopressin, glucocorticoid and thyroid hormone replacement, and LCH-III–based chemotherapy with prednisone and vincristine. Mercaptopurine (6-MP) was omitted due to hepatic dysfunction. However, chemotherapy was complicated by hepatic dysfunction, osteoporosis, and type 2 diabetes, which require intermittent cessation of chemotherapy and hepatoprotective therapy.

**Outcomes:**

Tumor size decreased to 1.0 × 1.2 × 0.7 cm; endocrine deficits persisted, requiring lifelong hormone replacement. The patient’s condition stabilized through continuous multidisciplinary care.

**Lessons:**

This case emphasizes that chronic endocrine symptoms warrant prompt pituitary imaging and early molecular testing, including BRAFV600E analysis, to avoid prolonged diagnostic delays and enable tailored therapy – especially in patients with comorbidities limiting standard treatment regimens.

## 1. Introduction

Langerhans Cell Histiocytosis (LCH) is a rare, complex, and clinically diverse disorder characterized by the abnormal proliferation of Langerhans cells, typically affecting multiple organ systems, including the pituitary gland. Pituitary involvement frequently manifests clinically as diabetes insipidus, panhypopituitarism, and various secondary endocrine deficiencies.^[1]^

While LCH exhibits a higher incidence in pediatric populations, its occurrence and clinical implications in adults, particularly regarding pituitary involvement, have increasingly garnered attention owing to diagnostic complexities and variable therapeutic responses.^[2]^ Despite advancements in diagnostic modalities and therapeutic strategies, literature reports indicate that the average interval from symptom onset to definitive diagnosis is approximately 8 months. In contrast, the considerably prolonged diagnostic delay experienced by this patient underscores the significant clinical challenges associated with this rare disease.^[3,4]^

The case described herein illustrates an atypically prolonged clinical presentation of pituitary LCH in a 38-year-old female patient whose diagnostic journey extended over approximately ten years. Initially presenting with symptoms characteristic of central diabetes insipidus, including excessive thirst and polyuria, the patient was later diagnosed with panhypopituitarism, including secondary adrenal insufficiency, hypothyroidism, and hypogonadotropic hypogonadism. This case is further distinguished by the coexistence of significant comorbidities, such as type 2 diabetes mellitus, osteoporosis, hyperlipidemia, gouty arthritis, and hepatic dysfunction, adding layers of complexity to diagnosis, therapeutic planning, and ongoing management. In the present case, BRAFV600E positivity was identified through molecular testing; however, targeted inhibition was not pursued, and management relied solely on conventional chemotherapy and supportive endocrine therapy.

A meticulous literature review was conducted utilizing academic databases including PubMed, Medline, and Embase, employing search terms such as “LCH,” “pituitary involvement,” “central diabetes insipidus,” “panhypopituitarism,” and “adult onset LCH” (Table 1). This comprehensive review underscores the uniqueness of this case, highlighting significant discrepancies between reported cases and the unusual patient.^[5–9]^ This case highlights key gaps in the literature on adult-onset pituitary LCH, particularly regarding its prolonged diagnostic course and complex clinical presentation. Unlike more acute pediatric cases, this patient experienced a decade-long diagnostic delay, with symptoms gradually progressing, which contrasts with the typical rapid onset described in most studies. Furthermore, the patient’s extensive endocrine dysfunction, such as central diabetes insipidus and hypogonadism, broadens the clinical understanding of LCH in adults, offering a unique presentation compared to previous cases typically documenting isolated symptoms. Additionally, the therapeutic challenges in this case, where chemotherapy was adjusted due to hepatic dysfunction, underscore the importance of individualized treatment plans for adults with comorbidities. Overall, this case contributes valuable insights into the indolent progression of adult-onset pituitary LCH and highlights the diagnostic and therapeutic complexities associated with multisystem involvement. The identification of the BRAFV600E mutation via molecular testing, confirmed through histopathological biopsy, firmly aligns this patient’s case within the spectrum of LCH, offering critical insights into potential genetic markers predictive of disease progression and therapeutic responsiveness.

**Table 1:** Case reports on pituitary and multisystem langerhans cell histiocytosis: diagnostic delays, BRAF-targeted therapy, and long-term outcomes.

Literature information	Case background	Diagnostic information	Treatment and intervention	Results and follow-up	Discussion and implications
Langerhans cell histiocytosis limited to the pituitary-hypothalamic axis – two case reports	Demographics: 14F and 9F, History: No prior comorbidities, Presentation: Polydipsia, polyuria, CDI, and mild hypopituitarism. MRI showed pituitary stalk masses.	Clinical: CDI, hypopituitarism, Imaging: MRI – isointense T1/hyperintense T2 pituitary stalk lesions, Pathology: Granulomas with eosinophils and CD1a/S100 + Langerhans cells, Differential: Germinoma, Final Diagnosis: Pituitary LCH (ICD: C96.6)	Treatment: Surgical biopsy + low-dose radiotherapy (20 Gy), Response: Mass reduction on MRI, Adjustments: None required	Short-term: Stable CDI, Long-term: Partial endocrine recovery and ongoing management (8-year follow-up)	Uniqueness: Rare localization; mimics germinoma, Significance: Reinforces biopsy necessity for pituitary masses, Limitations: Pediatric cases; limited BRAF testing, Recommendation: Early biopsy for atypical pituitary lesions
Langerhans cell histiocytosis: 2 case reports in adults and review of the literature.	Demographics: Adult patients (ages unspecified), History: Multisystem involvement, Presentation: Chronic symptoms (fatigue, bone pain, skin lesions)	Clinical: Bone lesions, skin rash, CDI, Imaging: CT/MRI – osteolytic lesions, pituitary enlargement, Pathology: CD1a + histiocytes, Differential: Metastasis, lymphoma, Final Diagnosis: Multisystem LCH (ICD: C96.6)	Treatment: Chemotherapy (vinca alkaloids, steroids), desmopressin, Response: Partial remission, Adjustments: Tailored for toxicity	Short-term: Symptom relief, Long-term: Persistent CDI (2 + years)	Uniqueness: Adult-onset multisystem LCH, Significance: Highlights delayed diagnosis in adults, Limitations: No molecular data, Recommendation: Systematic evaluation of multisystem complaints
Langerhans cell histiocytosis in old subjects: 2 rare case reports	Demographics: 71F and 77F, History: Chronic oral/genital lesions, Presentation: Mandibular osteolysis (Case 1), vulvar/palatal lesions (Case 2)	Clinical: Painful oral lesions, osteolysis, Imaging: CT – mandibular radiolucency, Pathology: CD1a+/S100 + histiocytes, Differential: Osteomyelitis, SCC, Final Diagnosis: Oral/vulvar LCH (ICD: C96.6)	Treatment: Case 1: Surgery; Case 2: Cladribine, Response: Complete remission (Case 2), Adjustments: Switched to cladribine after failed etoposide/radiotherapy	Short-term: Partial remission, Long-term: No recurrence (3 + years)	Uniqueness: Elderly presentation; atypical mucosal involvement, Significance: Cladribine efficacy in refractory cases, Limitations: No BRAF analysis, Recommendation: Immunohistochemistry for mucosal lesions
Solitary cranial Langerhans cell histiocytosis: Two case reports	Demographics: Unspecified adults, History: Skull trauma, Presentation: Skull masses with orbital erosion	Clinical: Asymptomatic skull lesions, Imaging: CT – osteolytic skull lesions, Pathology: CD1a+/S100 + histiocytes with eosinophils, Differential: Eosinophilic granuloma, Final Diagnosis: Unifocal cranial LCH (ICD: C96.6)	Treatment: Surgical resection + steroids/chemotherapy, Response: Lesion regression, Adjustments: None	Short-term: Stable, Long-term: Disease-free (7-year follow-up)	Uniqueness: posttraumatic cranial LCH, Significance: Supports combined surgical/medical management, Limitations: Limited molecular data, Recommendation: Trauma history as potential risk factor
Targeted therapy with vemurafenib in Brazilian children with refractory LCH	Demographics: Pediatric patients (unspecified ages), History: Multisystem refractory LCH, Presentation: High-risk organ involvement (liver, bone marrow)	Clinical: Hepatosplenomegaly, cytopenia, Imaging: PET-CT – multifocal lesions, Pathology: BRAF V600E mutation confirmed, Differential: Leukemia, Final Diagnosis: BRAF + multisystem LCH (ICD: C96.6)	Treatment: Vemurafenib (BRAF inhibitor), Response: Sustained remission, Adjustments: Reintroduced vemurafenib after relapse	Short-term: Rapid remission, Long-term: Disease control (3 + years)	Uniqueness: First BRAF-targeted therapy in Brazilian LCH, Significance: Validates vemurafenib for refractory cases, Limitations: Pediatric focus; unknown long-term effects, Recommendation: routine BRAF testing in refractory LCH

This table summarizes 5 pivotal case reports (2000–2024) addressing pituitary Langerhans cell histiocytosis (LCH) and its related multisystem manifestations. Selected for their relevance in diagnostic challenges, molecular innovations (BRAF V600E), and therapeutic responses, these cases collectively emphasize delayed recognition in adults, atypical endocrine presentations, and evolving treatment paradigms. Pediatric and adult reports were prioritized to contrast age-specific management strategies, while molecularly characterized cases highlight advancements in precision medicine. The limitations include small sample sizes and heterogeneous follow-up durations. This synthesis advocates for early immunohistochemical/molecular testing in protracted pituitary dysfunction and validates BRAF inhibitors as salvage therapy, bridging historical and contemporary LCH management approaches.

Adult-onset pituitary LCH is a rare condition that requires careful consideration, especially due to its subtle and slowly progressing endocrine abnormalities. Clinicians should suspect this diagnosis early in patients with persistent central diabetes insipidus, progressive anterior pituitary hormone deficiencies, or unexplained multisystem issues. Recognizing these signs early can prevent diagnostic delays, allow for timely histopathological confirmation, and enable prompt treatment before irreversible pituitary damage occurs. This report highlights the importance of clinical vigilance and early intervention in managing prolonged endocrine dysfunction, underscoring the critical role of endocrinologists in effectively addressing these complex cases.

## 2. Case presentation

A 38-year-old married female patient presented with a 10-year history of fatigue, excessive thirst, polyuria, and irregular menstruation. Approximately ten years prior, she began experiencing polydipsia and polyuria without identifiable triggers. The patient expressed frustration and anxiety regarding the persistent symptoms, which were ignored due to her lack of understanding of the disease’s potential severity. The delay significantly hindered timely diagnosis and intervention.

In September 2011, laboratory results showed mild fluctuations in immune markers (WBC count of 6.43 × 10^9^/L, neutrophils 44.5%, lymphocytes 48.7%), with normal liver function (ALT 18 U/L). Urinalysis revealed no significant abnormalities.

In April 2012, the patient underwent a pituitary plain and contrast-enhanced MRI examination using a PHILIPS 1.5T MRI, which showed that the pituitary gland had a vertical diameter of 0.7 cm with slightly uneven signals, and dynamic enhanced scanning revealed uneven enhancement; no abnormalities were found in the relevant structures of the sellar region, and the tentative diagnosis suggested that pituitary microadenoma could not be excluded, with a recommendation for reexamination. Meanwhile, the results of the sex hormone test in the same month showed that follicle-stimulating hormone, pituitary prolactin (PRL) and testosterone (TSTO) and other related indicators were all within the normal range, suggesting no significant abnormalities in the endocrine sex hormone axis (Table 2).

**Table 2:** Summary of endocrine abnormalities over time.

Indicator Category	Abbreviation	Reference Range	2012.04	2023.02	2023.11	2024.01	2025.06
Sex Hormones	FSH	3.85–8.78mIU/mL in the Follicular phase, 4.54–22.51 mIU/mL in the Ovulatory phase, and 1.79–5.12 mIU/mL in the Luteal phase	2.51	1.29↓	0.85↓	–	0.62↓
Sex Hormones	LH	2–12 IU/L in the Follicular phase, 14–96 IU/L in the Ovulatory phase, and 1–15 IU/L in the Luteal phase	6.53	0.13↓	<0.10↓	–	<0.10↓
Sex Hormones	PRL	3.34–26.72 ng/mL	12.42	3.13↓	2.94↓	–	2.26↓
Sex Hormones	TSTO	14–76 ng/dL	20.50	–	–	–	9.80↓
Thyroid Function	TSH	0.35~5.5 μIU/mL	2.35	3.094	0.080↓	0.052↓	0.031↓
Thyroid Function	T3	3.5~6.5 pmol/L	3.89	3.25↓	3.50	3.62	4.49
Thyroid Function	T4	11.5~22.7 pmol/L	17.67	6.83↓	12.37	11.57	12.71
Cortisol	Cor	52~350 ng/mL	–	15.1↓	–	–	16.4↓
Cortisol	Cor (8am)	72.6–322.8 ng/mL	–	21.8↓	–	–	17.8↓
Cortisol	Cor (4pm)	32.4~150.0 ng/mL	–	11.4↓	–	–	8.5↓
Cortisol	24 h Urinary Cortisol	30~350 ug/24H	–	28.0↓	–	–	20.2↓

“↓” indicates that the detected value is lower than the reference range. Blank cells mean no corresponding test data was reported.

In 2013, the patient developed polydipsia and polyuria without identifiable triggers, with daily water intake and urine output reaching 3000 to 4000 mL. Following a water deprivation and vasopressin test at our hospital, she was diagnosed with central diabetes insipidus. Despite initial symptomatic relief with desmopressin acetate (0.1 mg twice daily), the patient independently reduced the dosage, without regular follow-up or adjustments, leading to a worsening of symptoms and increasing frustration with her management.

In February 2023, the patient presented to our Endocrinology Department with a 10-year history of fatigue, thirst, polyuria, and irregular menstruation, and 1-year memory decline, accompanied by significant memory impairment and sluggish responses. Laboratory tests revealed severe hypernatremia. Further evaluation confirmed adrenal insufficiency, secondary hypothyroidism, and concomitant hypogonadotropic hypogonadism (Table 2). Ultimately, the patient was diagnosed with panhypopituitarism. Dynamic contrast-enhanced MRI of the pituitary region identified an irregular soft tissue mass at the junction of the optic chiasm and pituitary stalk, measuring approximately 1.9 cm × 1.4 cm × 1.2 cm, suggesting possible diagnoses of craniopharyngioma, germ cell tumor, or LCH. Neurosurgery recommended biopsy, but the patient refused due to “due to concerns about surgical risks” and was temporarily started on hormone replacement therapy (desmopressin acetate, prednisone, levothyroxine sodium).

In June 2023, the patient visited a hospital in Wuhan, where a germ cell tumor was excluded (a biopsy was not performed). However, due to ongoing concerns about surgery, the patient declined further treatment and continued with the original hormone replacement regimen. Despite symptom management with hormone therapy, she expressed considerable anxiety about the long-term implications of her disease.

In November 2023, the patient underwent nasal saddle area biopsy at a tertiary hospital in Shanghai under general anesthesia. Gross examination revealed grayish-white fragmented tissue with a maximum diameter of 0.5 cm. Microscopic examination showed chronic inflammation with sparse histiocytic proliferation. Immunohistochemical results were as follows: S100 (partial +), CD1a (individual +), CD68 (partial +), CD3 (partial +), CD20 (partial +), CD138 (few +), Ki67 (6% partial +), GFAP (few +), CD117 (individual +), Syn (few +). Pathological diagnosis: Chronic inflammation with sparse histiocytic formation in the hypothalamic-pituitary region, with occasional CD1a positivity. Molecular testing using ARMS-PCR confirmed the BRAFV600E mutation, a sensitive method for detecting point mutations, thereby diagnosing LCH, with tumor cells constituting over 30% of the specimen. Special stains showed PAS (−), silver stain (−), and acid-fast stain (−), further supporting the diagnosis of LCH.

**Figure 1.**
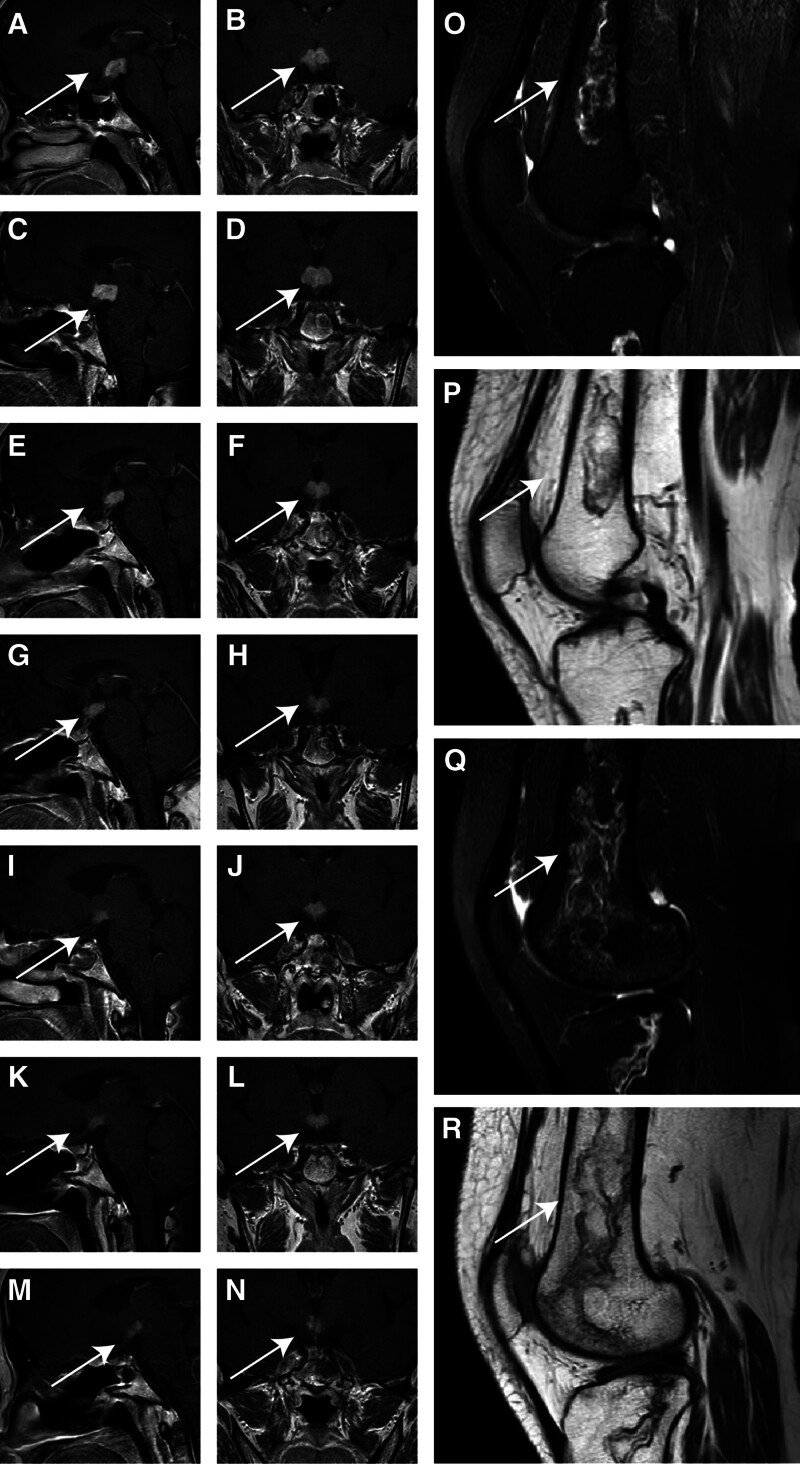
MRI of the serial pituitary and knee joints from 2023 to 2024. (A) to 1N show the patient’s pituitary sella region MRI scans. (A and B) are from February 2023, (C and D) are from January 2024, Figures (E and F) are from March 2024, (G and H) are from April 2024, (I and J) are from June 2024, (K and L) are from September 2024 and (M and N) are from June 2025. The arrows indicate the tumor mass. As shown in the images, the tumor mass gradually shrinks, with little change in size between June 2024 and June 2025. Figures 1O to 1R show the patient’s knee joint MRI scans. The arrows indicate the areas of bone infarction, with abnormal signals in the distal femurs and proximal tibias on both sides, suggesting femoral infarction.

The patient was initially discharged with an adjusted medication regimen that included hydrocortisone acetate tablets (25 mg in the morning, 12.5 mg in the afternoon), desmopressin acetate (0.1 mg twice daily), and levothyroxine sodium (50 µg daily), supplemented with hepatoprotectants, calcium, and vitamin D3. However, in January 2024, she was readmitted due to the onset of back and leg pain, fatigue, and generalized malaise. A bilateral knee MRI revealed bone infarctions in both femoral and tibial regions. Prior to initiating chemotherapy, a pituitary MRI showed a lesion size of 1.9 × 1.7 × 1.1 cm, slightly increased from 1.9 × 1.4 × 1.2 cm in February 2023 (Fig. 1). Additionally, the patient reported an exacerbation of fatigue symptoms compared to earlier.

Her medical history included fatty liver disease with abnormal hepatic function, hyperlipidemia, gouty arthritis, and type 2 diabetes, the latter being treated with acarbose (50 mg 3 times daily). She also had osteoporosis, for which she was treated with calcium carbonate, vitamin D3, and alendronate sodium. She denied any history of hypertension, coronary artery disease, cerebrovascular disease, hepatitis B, tuberculosis, typhoid, or drug and food allergies and had undergone routine national immunization. She was employed locally and had no exposure to endemic areas. The patient denied smoking, alcohol consumption, and drug abuse. Her menstrual history was notable for menarche at a typical age, marriage at 23, a successful pregnancy and childbirth, and premature menopause at 25 years of age.

Physical examination upon admission showed a stable general condition with clear consciousness and normal vital signs, including a temperature of 36.5°C, pulse rate of 55 beats per minute, respiration rate of 18 breaths per minute, and blood pressure of 128/84 mm Hg. No eyelid edema, visual disturbances, or palpable lymphadenopathy were noted. The skin was dry and rough, with poor elasticity, sparse eyebrows, and loss of pubic and axillary hair. Respiratory and cardiovascular examinations were unremarkable, and abdominal examination revealed no tenderness or palpable mass. Neurological examination showed reduced reflexes and diminished pulses in the lower limbs.

Laboratory investigations revealed abnormal electrolyte levels, including elevated sodium (155.7–157.5 mmol/L), chloride (113.6–119.1 mmol/L), and carbon dioxide (30.1–33.5 mmol/L). Thyroid function tests consistently showed low TSH levels and low-to-normal thyroid hormone levels. Cortisol rhythm tests indicated low cortisol levels, suggesting adrenal insufficiency. Sex hormone testing revealed notably low follicle-stimulating hormone and luteinizing hormone levels, indicating hypogonadotropic hypogonadism (Table 2). During treatment, significant alterations in endocrine function were noted, as detailed in Table 2, which includes the patient’s dynamic hormone profiles reflecting changes in thyroid function, sex hormone levels, adrenal function, and gonadotropin levels.

Pituitary MRI scans revealed a mass lesion initially measuring 1.9 cm × 1.7 cm × 1.1 cm at the optic chiasm-mammillary body junction. posttreatment imaging in March and April 2024 demonstrated a progressive reduction in tumor size to 1.4 cm × 1.4 cm × 0.9 cm and further to 1.2 cm × 1.4 cm × 0.7 cm, respectively. In June 2024, a bilateral knee MRI revealed bone infarctions in the femoral and tibial regions, along with meniscal degeneration and joint effusion. By September 2024, the pituitary lesion had stabilized at approximately 1.0 cm × 1.2 cm × 0.7 cm, indicating partial remission (Fig. 1).

Initial treatment consisted of systemic chemotherapy according to the LCH-III protocol, which included prednisone (40 mg/m^2^ daily, reduced after the initial dosing), vincristine (1.5 mg/m^2^ weekly), and planned mercaptopurine therapy. However, chemotherapy was intermittently disrupted due to hepatic dysfunction, marked by elevated liver enzyme levels, requiring temporary cessation and hepatoprotective interventions. Despite an initial positive response with tumor reduction, chemotherapy was halted due to significant adverse symptoms, including abdominal discomfort and profound fatigue, as outlined in Table 3. The patient’s subsequent management included continued hormone replacement therapy with prednisone, desmopressin acetate, and levothyroxine sodium.

**Table 3:** Detailed chemotherapy regimen from January 13, 2024, to May 31, 2024.

Date(s)	Medication	Theoretical Dose	Actual Dose	Remarks
January 13, 24	Vincristine	1.5 mg/m²	2.0 mg	Chemotherapy initiation
January 13–January 19, 2024	Prednisone	40 mg/m²	50 mg	Administered once daily
January 20, 2024	Vincristine	1.5 mg/m²	2.0 mg	–
January 21– January 31, 2024	Prednisone	40 mg/m²	50 mg	Once daily; elevated AST 189 U/L, ALT 114 U/L on Jan 26. Treatment delayed until liver protection therapy normalized levels (AST 44 U/L, ALT 32 U/L) on Feb 1
February 1, 2024	Vincristine	1.5 mg/m²	2.0 mg	–
February 1–February 29, 2024	Prednisone	40 mg/m²	50 mg	Once daily
March 1, 2024	Vincristine	1.5 mg/m²	2.0 mg	Elevated AST 63 U/L, ALT 112 U/L on Feb 22 delayed treatment; resumed after levels improved (AST 58 U/L, ALT 92 U/L) on Mar 1
March 1–March 8, 2024	Prednisone	40 mg/m²	50 mg	Once daily
March 9, 2024	Vincristine	1.5 mg/m²	2.0 mg	–
March 9–March 16, 2024	Prednisone	40 mg/m²	50 mg	Once daily; dose reduced by 10 mg every 3 days to 30 mg daily starting Mar 10
March 17, 2024	Vincristine	1.5 mg/m²	2.0 mg	–
March 17–March 23, 2024	Prednisone	40 mg/m²	50 mg	Once daily; dose reduction as above, starting Mar 18
March 24, 2024	Vincristine	1.5 mg/m²	2.0 mg	–
March 24–March 29, 2024	Prednisone	40 mg/m²	50 mg	Once daily; dose reduced to 30 mg starting Mar 27
March 30, 2024	Vincristine	1.5 mg/m²	2.0 mg	–
March 30–April 6, 2024	Prednisone	40 mg/m²	50 mg	Once daily; dose reduced to 30 mg starting Apr 2
April 7, 2024	Vincristine	1.5 mg/m²	2.0 mg	–
April 7–April 13, 2024	Prednisone	40 mg/m²	50 mg	Once daily; dose reduced to 30 mg starting Apr 10
April 14, 2024	Vincristine	1.5 mg/m²	2.0 mg	
April 14–April 22, 2024	Prednisone	40 mg/m²	50 mg	Once daily; dose reduced to 30 mg starting Apr 17
April 23, 2024	Vincristine	1.5 mg/m²	2.0 mg	–
April 23–April 29, 2024	Prednisone	40 mg/m²	50 mg	Once daily; dose reduced to 30 mg starting Apr 26
April 30, 2024	Vincristine	1.5 mg/m²	2.0 mg	–
Apr 30–May 6, 2024	Prednisone	40 mg/m²	50 mg	Once daily; dose reduced to 30 mg starting May 3
May 7, 24	Vincristine	1.5 mg/m²	2.0 mg	–
May 7–May 26, 2024	Prednisone	40 mg/m²	50 mg	Once daily; dose reduced to 30 mg starting May 10
May 27, 2024	Vincristine	1.5 mg/m²	2.0 mg	Every 3 wk
May 27–May 31, 2024	Prednisone	40 mg/m²	50 mg	Once daily for 5 d; discontinued due to patient-reported fatigue and limb numbness; mercaptopurine (6-MP) was not administered

This table summarizes the chemotherapy regimen for the patient, highlighting adjustments made due to hepatic dysfunction and adverse reactions. Mercaptopurine (6-MP) was excluded due to significant hepatotoxic risk.

**Figure 2.**
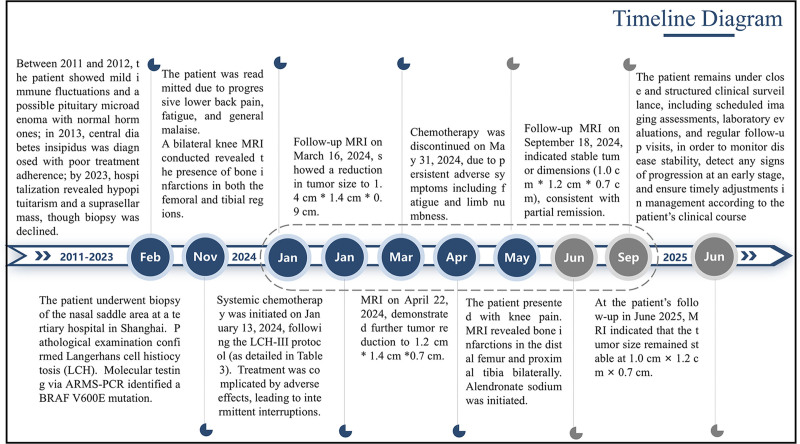
Timeline Diagram. Showing the timeline of the patient’s diagnosis and treatment from 2011 to 2025.

**Figure 3.**
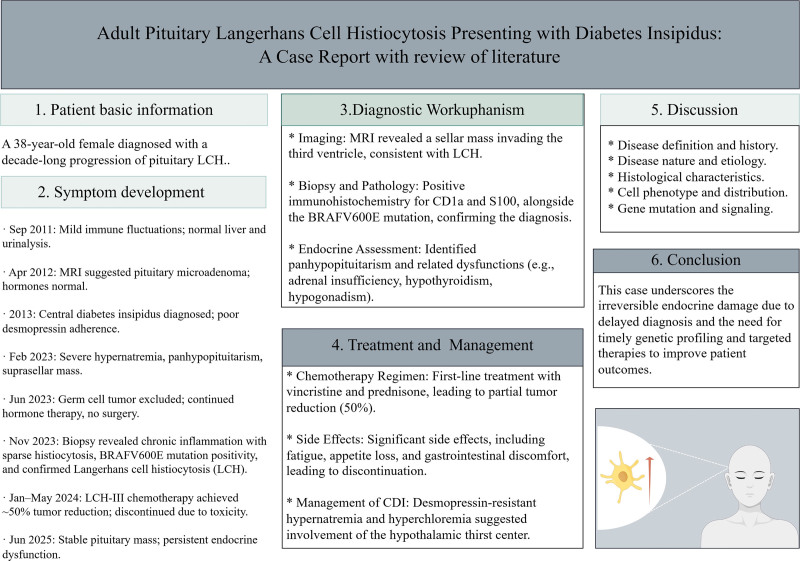
Graphic abstract. Graphical abstract illustrating the patient’s chief complaint, diagnosis, treatment course, and prognosis.

At the latest follow-up, in June 2025, the patient’s pituitary mass remained stable at a size of 1.0 cm × 1.2 cm × 0.7 cm (Fig. 1), with no further progression in lesion size or persistent endocrine dysfunction. This warranted ongoing endocrinological follow-up and supportive management (Figs. 2 and 3).

Although radiological follow-up demonstrated significant regression of the pituitary mass, the patient continued to experience irreversible endocrine deficits that required lifelong hormone replacement. Incorporating structured evaluations of functional capacity and quality of life into the follow-up of pituitary LCH would provide a more complete picture of therapeutic benefit and long-term prognosis, especially when endocrine disturbances persist despite apparent tumor control.

## 3. Discussion

This case of adult-onset pituitary LCH presents a rare, prolonged clinical course with gradual endocrine dysfunction,^[10,11]^ contrasting with typical rapid progression seen in other cases.^[12]^

**Figure 4.**
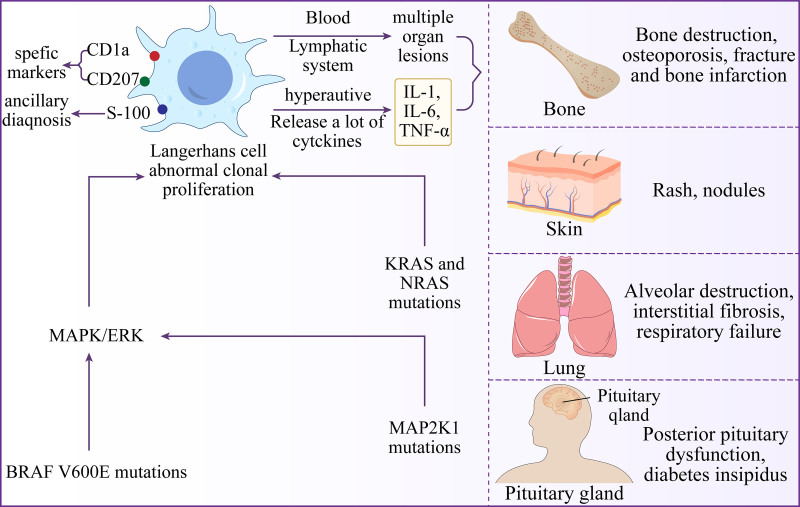
Pathogenesis of LCH. Gene mutations including BRAF V600E and MAP2K1, lead to abnormal proliferation of Langerhans cells. The positive expression of cell surface markers CD1a and CD207 represents the abnormal proliferation of LC cells. These abnormally proliferating cells accumulate throughout various systems in the body, such as the skeletal and respiratory systems, via the blood and lymphatic systems. LCH = Langerhans cell histiocytosis.

From an endocrine perspective, the identification of the BRAFV600E mutation is particularly significant (Fig. 4) as it provides a direct molecular link and potential therapeutic target.^[13]^ The BRAFV600E mutation drives the abnormal proliferation of Langerhans cells through MAPK pathway activation, thereby influencing tumor growth and endocrine dysfunction. In patients with LCH, the prevalence of BRAF V600E mutations varies by age, with a higher mutation detection rate observed in pediatric patients compared to adults.^[14]^ Previous literature has demonstrated that 47% of adult LCH cases (age > 18 years) harbor V600E-positive lesions, whereas the positivity rate is 53% in pediatric cases (age < 18 years).^[15]^ The literature frequently highlights BRAFV600E’s prognostic and therapeutic implications, reinforcing targeted molecular diagnostics as critical for timely intervention.^[14]^ From a prognostic standpoint, the BRAF V600E mutation is frequently associated with an increased risk of recurrence, whereas early targeted intervention can substantially mitigate this risk.^[16]^ Specifically, BRAF inhibitors (e.g., vemurafenib, dabrafenib) have demonstrated high efficacy in BRAFV600E-mutant LCH, achieving rapid remission in refractory multisystem disease.^[17,18]^ For patients like ours with chemotherapy complications (hepatotoxicity, osteoporosis), BRAF/MEK inhibitors offer a less toxic alternative to conventional chemotherapy and may mitigate long-term morbidities.^[19,20]^ Furthermore, molecular subtyping of LCH – distinguishing BRAF, MAP2K1, or ARAF alterations – can guide precision therapy, underscoring the need for routine molecular profiling in adult LCH Molecular markers and miRNA pathway insights support precision therapy, underscoring their practical value for tailored treatment strategies in rare diseases.^[21–23]^ Dynamic network biomarkers elucidate molecular heterogeneity in cancer.^[24]^ However, despite the confirmed BRAF positivity, vemurafenib was not administered in this case because of the patient’s economic constraints, as the high cost of the drug made it unaffordable for her. Management therefore relied solely on conventional chemotherapy and supportive endocrine therapy.

The standard LCH-III protocol typically includes prednisone, vinblastine (VBL) and mercaptopurine (6-MP).^[25]^ Notably, 6-MP was omitted from the therapeutic plan because of significant hepatic dysfunction, as evidenced by elevated liver enzymes during the initial chemotherapy cycles. This exclusion is clinically justified, as 6-MP is metabolized hepatically, thereby posing an additional risk of hepatotoxicity. Thus, regimen modification is imperative to balance treatment efficacy with patient safety.^[26]^ The choice of an individualized treatment strategy in this case was based on a comprehensive assessment of the patient’s comorbidities and treatment tolerance. The patient had a history of fatty liver with abnormal liver function, and during the early stages of treatment, chemotherapy induced elevations in AST (up to 189 U/L) and ALT (up to 114 U/L), placing her at high risk of 6-MP–related hepatotoxicity. Pharmacokinetic factors are critical: 6-MP is metabolized in the liver via xanthine oxidase and thiopurine methyltransferase (TPMT), and impaired hepatic function may reduce drug clearance, leading to the accumulation of toxic metabolites.^[26]^ Given the patient’s baseline hepatic vulnerability, the risk of severe liver injury associated with 6-MP, such as fulminant hepatitis or worsening cirrhosis, outweighed its potential benefits. This decision aligns with recent guidelines, which emphasize that in the treatment of LCH, chemotherapy dosing should be adjusted based on comorbidities, particularly for drugs with organ-specific toxicities.^[3]^ Despite these complications, the patient showed a reduction in tumor size and stabilization of her condition, highlighting the potential of chemotherapy, albeit with the need for individualized adjustments based on the patient’s comorbidities and response to treatment.^[26]^

The persistent hypernatremia and hyperchloremia during desmopressin therapy in the context of LCH may reflect complex underlying pathophysiological mechanisms. Desmopressin, typically used for central diabetes insipidus, acts by increasing water reabsorption in the kidneys.^[27]^ However, LCH-related pituitary dysfunction could alter renal handling of electrolytes, impairing sodium and chloride regulation. Additionally, concurrent endocrine deficiencies, such as adrenal insufficiency, may contribute to disrupted electrolyte balance, necessitating careful monitoring and adjustment of treatment.^[28]^

A significant event during management was bone infarction detected by bilateral knee MRI in June 2024. The necessity of monitoring skeletal complications in LCH, which may result from direct disease-related bone infiltration and infarction, prolonged glucocorticoid therapy, or chemotherapeutic agents.^[29]^ Cytokines like IL-1, IL-6, and TNF-α drive osteoclastogenesis and bone resorption in LCH through inflammatory pathways.^[30]^ In this case, this patient experienced a femoral infarction prior to steroid initiation supports a primary contribution from LCH-associated bone disease, although subsequent lesions could be compounded by treatment effects.

In this patient’s case, it points to a possible underlying endocrine pathology, which might be linked to the pituitary dysfunction due to LCH. Premature menopause is typically associated with primary ovarian insufficiency, autoimmune disorders, genetic conditions, or chemotherapy/radiation exposure.^[31]^ It is uncommon for a patient to experience successful reproduction, followed by premature menopause, as the hormonal milieu during pregnancy might have temporarily compensated for underlying ovarian dysfunction. The premature menopause observed in this patient could have been triggered by the progressive pituitary damage caused by LCH, which resulted in a dysfunction of the hypothalamic-pituitary-gonadal axis, leading to early ovarian failure.

The limitations of this report pertain to its singular nature, thereby inherently restricting generalized conclusions. The ten-year diagnostic delay in this adult LCH patient can be attributed to 3 interconnected factors: insufficient awareness of adult-onset LCH among clinicians, often leading to endocrine symptoms being misattributed to more common conditions; the slow onset and nonspecific nature of symptoms, resulting in initial misdiagnosis and delayed referral; early imaging often reveals only mild thickening of the pituitary stalk, and biopsy, the gold standard for diagnosis, is frequently postponed in stable patients due to its invasiveness. To address these issues, we propose the following strategies: develop an endocrine “danger signal” checklist; MRI should be considered earlier, even when symptoms seem nonspecific, to identify any structural pituitary abnormalities. For patients already diagnosed with LCH, long-term follow-up should include structured monitoring: assess endocrine function quarterly; perform pituitary MRI every 6 months for the first 2 years (and annually thereafter)^[22,23]^; monitor bone density every 6 months; liver function monthly during the first 6 months post-chemotherapy; screen for diabetes and hyperlipidemia annually; and assess quality of life using questionnaires. The focus of management should be on optimizing hormone replacement, controlling comorbidities, and early detection of recurrence, with targeted therapy reserved for disease progression.

Additionally, intermittent chemotherapy discontinuation complicates the precise interpretation of clinical outcomes. Variation in clinical outcomes could have been influenced by the unique patient compliance, treatment interruptions due to adverse effects, and individualized response to therapy, all of which require cautious interpretation.

This case describes adult-onset pituitary LCH with BRAF V600E mutation, notable for a prolonged prediagnostic course, irreversible panhypopituitarism, and chemotherapy-related hepatic dysfunction. The Naranjo nomogram indicated a probable association between chemotherapy and hepatotoxicity, necessitating treatment modification.^[32]^ Despite marked lesion regression, the absence of hormonal recovery suggests destructive damage to the pituitary stalk and neurohypophysis rather than reversible inflammatory changes. This case underscores the need for early recognition of persistent endocrine dysfunction, timely pituitary imaging, and molecular testing to facilitate intervention before irreversible injury occurs. Furthermore, it highlights the importance of individualized treatment strategies, particularly in patients with hepatic comorbidities, and reinforces the value of a multidisciplinary approach in optimizing outcomes and minimizing morbidity in adult-onset pituitary LCH.

## 4. Conclusion

This case underscores the critical importance of early clinical suspicion and the integration of advanced diagnostic tools in the management of rare diseases like adult-onset pituitary LCH. The patient’s decade-long diagnostic delay emphasizes the need for increased vigilance when patients present with chronic, nonspecific endocrine disturbances such as polyuria, polydipsia, and fatigue, which can mask more complex pathologies. The delayed diagnosis resulted in prolonged suffering and complicated treatment, leading to irreversible pituitary dysfunction despite eventual intervention. Future research should focus on identifying biomarkers predictive of atypical disease courses, optimizing diagnostic strategies, and refining therapeutic protocols for the complex presentations of pituitary LCH.

## Acknowledgments

The authors wish to express their gratitude to the patient described in this case report for her invaluable contribution to medical research. We sincerely appreciate her informed consent to share clinical details and imaging studies, as well as her consistent cooperation throughout the extensive diagnostic and therapeutic processes. Her willingness to participate in this publication provided essential insights into the management of adult-onset multisystem LCH. The authors gratefully acknowledge the use of Figdraw for assistance in figure preparation.

## Author contributions

**Conceptualization:** Yiwei Hou, Yu Yang, Wufei Zhu.

**Data curation:** Yiwei Hou, Yu Yang, Wufei Zhu.

**Formal analysis:** Yiwei Hou, Yu Yang, Wufei Zhu.

**Funding acquisition:** Yu Yang, Xiangyu Liao, Shixin Li.

**Investigation:** Yiwei Hou, Yu Yang, Xiangyu Liao, Yunxi Fu, Mingxu Tong.

**Methodology:** Yiwei Hou, Yu Yang, Xiangyu Liao, Beihan Li, Mingxu Tong.

**Project administration:** Yiwei Hou, Yu Yang, Beihan Li.

**Resources:** Yiwei Hou, Yu Yang, Li Yi, Beihan Li, Yunxi Fu.

**Software:** Yiwei Hou, Yu Yang, Xiangyu Liao, Li Yi, Shixin Li, Beihan Li, Yunxi Fu.

**Supervision:** Xiangyu Liao, Li Yi, Shixin Li.

**Validation:** Xiangyu Liao, Li Yi, Shixin Li.

**Visualization:** Xiangyu Liao, Li Yi, Zijun Zhou.

**Writing – original draft:** Yiwei Hou, Li Yi, Zijun Zhou, Wufei Zhu.

**Writing – review & editing:** Yiwei Hou, Yu Yang, Li Yi, Zijun Zhou, Wufei Zhu.
